# Plasma-derived parasitic microRNAs have insufficient concentrations to be used as diagnostic biomarker for detection of *Onchocerca volvulus* infection or treatment monitoring using LNA-based RT-qPCR

**DOI:** 10.1007/s00436-017-5382-5

**Published:** 2017-01-22

**Authors:** Ole Lagatie, Linda Batsa Debrah, Alex Debrah, Lieven J. Stuyver

**Affiliations:** 1 Janssen Diagnostics, Janssen R&D, Turnhoutseweg 30, 2340 Beerse, Belgium; 2 Kumasi Centre for Collaborative Research, Kwame Nkrumah University of Science and Technology, Kumasi, Ghana; 3 Faculty of Allied Health Sciences, Kwame Nkrumah University of Science and Technology, Kumasi, Ghana

**Keywords:** *Onchocerca volvulus*, River blindness, Onchocerciasis, Biomarker, miRNA, Diagnostic

## Abstract

River blindness, caused by infection with the filarial nematode *Onchocerca volvulus*, is a neglected tropical disease affecting millions of people. There is a clear need for diagnostic tools capable of identifying infected patients, but that can also be used for monitoring disease progression and treatment efficacy. Plasma-derived parasitic microRNAs have been suggested as potential candidates for such diagnostic tools. We have investigated whether these parasitic microRNAs are present in sufficient quantity in plasma of *Onchocerca*-infected patients to be used as a diagnostic biomarker for detection of *O. volvulus* infection or treatment monitoring. Plasma samples were collected from different sources (23 nodule-positive individuals and 20 microfilaridermic individuals), microRNAs (miRNAs) were extracted using Qiagen miRNeasy kit, and a set of 17 parasitic miRNAs was evaluated on these miRNA extracts using miRCURY Locked Nucleic Acid (LNA) Universal RT microRNA PCR system. Of the 17 miRNAs evaluated, only 7 miRNAs were found to show detectable signal in a number of samples: bma-miR-236-1, bma-miR-71, ov-miR71-22nt, ov-miR-71-23nt, ov-miR-100d, ov-bantam-a, and ov-miR-87-3p. Subsequent melting curve analysis, however, indicated that the signals observed for ov-miR-71 variants and ov-miR-87-3p are non-specific. The other miRNAs only showed positive signal in one or few samples with Cq values just below the cutoff. Our data indicate that parasitic miRNAs are not present in circulation at a sufficiently high level to be used as biomarker for *O. volvulus* infection or treatment monitoring using LNA-based RT-qPCR analysis.

## Introduction

Onchocerciasis, a neglected tropical disease commonly known as river blindness, is caused by infection with the filarial nematode, *Onchocerca volvulus* (*O. volvulus*). In Africa, at least 120 million people are at risk of infection (Borup et al. 2003; Enk 2006). The last comprehensive survey conducted in 2008 indicated that 26 million people were infected with *Onchocerca*, of whom 265,000 were blind and 746,000 were visually impaired. In addition, approximately 4 million people suffer from onchodermatitis with severe itching (WHO 2010). Since 1987, ivermectin has been used in mass drug administration (MDA) programs to treat hundreds of millions of people resulting in a reduction of both visual impairment and symptomatic onchodermatitis (Cupp et al. 2011).

Besides clinical examination by palpation of nodules formed by adult worms (macrofilariae), diagnostic tools for detection of *O. volvulus* infection traditionally were limited to detection of microfilariae (mf) in small, superficial skin biopsy samples (so-called skin snips) (Taylor et al. 1989). More recent research, however, also resulted in the development of rapid-format tests for the detection of IgG4 antibodies to the parasitic antigen Ov-16 (Chandrashekar et al. 1996; Golden et al. 2013; Lavebratt et al. 1994; Lipner et al. 2006; Steel et al. 2015; Weil et al. 2000). This test is predominantly useful in a surveillance setting where it can be used for the identification of incident infections in communities having already undergone MDA (Golden et al. 2013). Diagnostic tests that can distinguish between past and active infection or that can be used for monitoring disease progression or treatment response are currently still lacking. Several efforts have therefore been undertaken to identify novel biomarkers that hold potential to be used in such diagnostic tests (Vlaminck et al. 2015). For *Dirofilaria immitis* (heartworm) and *Wuchereria bancrofti* (the most prevalent cause of lymphatic filariasis), this has been successful as sensitive assays have been developed that detect circulating filarial antigens in the blood (More and Copeman 1990; Weil 1987; Weil et al. 2013; Weil et al. 1997). An approach that has shown promise is the use of metabolome analysis of serum or urine samples from infected individuals, which has led to the identification of urinary *N*-acetyltyramine-*O*,β-glucuronide (NATOG) as a unique biomarker for *O. volvulus* infection (Denery et al. 2010; Globisch et al. 2013; Lagatie et al. 2016).

Recent deep sequencing work has revealed the presence of many microRNA (miRNA) homologues in nematode parasites. Studies on whole worm extracts have identified miRNAs produced by *Brugia malayi* (Poole et al. 2010; Poole et al. 2014) and *D. immitis* (Fu et al. 2013). Other works suggest that these parasite miRNAs may play a role in adaptation to life in the vertebrate host (Britton et al. 2014) and possibly also modulate the innate immunity of the host (Buck et al. 2014). Indeed, deep sequencing studies identified several parasitic miRNAs in host circulation upon infection with *Loa loa* (Tritten et al. 2014b), *Onchocerca ochengi* (Tritten et al. 2014b), *Litomosoides sigmodontis* (Buck et al. 2014), *Schistosoma mansoni* (Hoy et al. 2014), *D. immitis* (Tritten et al. 2014a), and *O. volvulus* (Quintana et al. 2015; Tritten et al. 2014a).

*O. volvulus* miRNAs have been detected in serum of infected individuals (Quintana et al. 2015). Of the six different miRNAs detected (miR-71, lin-4, miR-100d, miR-87-3p, mir-100a, and bantam-a), only miR-71 and lin-4 were found both in samples of patients from Cameroon and in samples of patients from Ghana. MiR-87-3p and miR-100a were only detected in samples of Ghanaian patients. The other two miRNAs (miR100d and bantam-a) were found both in infected and uninfected patients from Ghana. This work was performed only with pooled serum using small RNA sequencing on the Illumina platform. No information was collected on the detection frequency of these miRNAs in serum of infected individuals, nor on the levels in individual samples. Also, no confirmation of these findings using standard PCR technology has been performed. Therefore, we wanted to determine the levels of several (putative) *O. volvulus* miRNAs in different sets of *O. volvulus*-infected individuals using Locked Nucleic Acid (LNA)-based RT-qPCR.

## Materials and methods

### Study samples

The following material was obtained through the Filariasis Research Reagent Resource Center (FR3), Division of Microbiology and Infectious Diseases, NIAID, NIH: a set of 20 *O. volvulus*-infected human serum samples, from Dr. Nutman, collected in Cameroon. All samples were de-identified before they were provided to FR3, and usage of these samples for research purposes was approved by the Smith College Institutional Review Board (IRB). Information on *O. volvulus* infection (number of microfilaria/mg skin and number of palpable nodules) was provided by FR3, along with demographic information (Table 1). All infected individuals had at least two palpable nodules and 25 mf/mg skin (microfilaridermia) as determined by skin snip. Sera were collected from clotted blood obtained by venipuncture. The negative panel was composed of eight serum samples from healthy, non-infected individuals from Southern Africa, was collected in FDA-regulated donor centers in the USA, and was provided by Tissue Solutions Ltd. (Glasgow, Scotland). Written informed consent was obtained from all individuals, and all samples were decoded and de-identified before they were provided for research purposes. Demographic information was also provided (Table 1). All samples were stored at −80 °C until analysis.

**Table 1:** Demographic information of study populations

Subject ID	Origin	Group	Age	Sex	mf/mg skin	No. of nodules	Ov16 IgG4	Source
852-005	Ghana	Nod.	54	M	0	2	−	KCCR
854-036	Ghana	Nod.	62	M	0	1	+	KCCR
853-043	Ghana	Nod.	74	M	0	1	+	KCCR
855-037	Ghana	Nod.	55	M	0	2	−	KCCR
850-011	Ghana	Nod.	21	F	0	2	+	KCCR
850-034	Ghana	Nod.	22	M	0	1	−	KCCR
852-025	Ghana	Nod.	28	F	0	1	−	KCCR
852-046	Ghana	Nod.	25	F	0	1	+	KCCR
854-009	Ghana	Nod.	58	M	0	1	+	KCCR
851-033	Ghana	Nod.	30	M	0	1	+	KCCR
855-040	Ghana	Nod.	50	F	0	3	+	KCCR
853-060	Ghana	Nod.	44	M	0	2	+	KCCR
854-030	Ghana	Nod.	40	F	0	2	−	KCCR
850-052	Ghana	Nod.	29	F	0	1	+	KCCR
856-020	Ghana	Nod.	52	M	0	5	+	KCCR
851-014	Ghana	Nod.	58	M	0	2	+	KCCR
853-051	Ghana	Nod.	58	F	0	1	−	KCCR
852-028	Ghana	Nod.	80	F	0	1	+	KCCR
852-038	Ghana	Nod.	21	M	0	1	−	KCCR
854-031	Ghana	Nod.	60	F	0	1	+	KCCR
854-043	Ghana	Nod.	67	M	0.6	4	+	KCCR
853-014	Ghana	Nod.	21	M	0	1	+	KCCR
850-072	Ghana	Nod.	28	M	0	1	+	KCCR
500-501	Ghana	NEC	26	M	0	0	−	KCCR
500-503	Ghana	NEC	25	F	0	0	−	KCCR
500-504	Ghana	NEC	43	M	0	0	−	KCCR
500-505	Ghana	NEC	26	M	0	0	−	KCCR
500-506	Ghana	NEC	28	F	0	0	−	KCCR
500-507	Ghana	NEC	28	M	0	0	−	KCCR
MC 179	Cameroon	mf	47	M	100	7	+	FR3
MC 202	Cameroon	mf	52	M	89	9	+	FR3
MC 211	Cameroon	mf	54	F	36	2	+	FR3
MC 215	Cameroon	mf	55	F	45	2	−	FR3
MC 224	Cameroon	mf	60	F	26	3	+	FR3
MC 226	Cameroon	mf	75	F	99	7	+	FR3
MC 234	Cameroon	mf	50	F	70	2	+	FR3
MC 253	Cameroon	mf	54	M	40	6	+	FR3
MC 260	Cameroon	mf	39	F	26	2	+	FR3
MC 319	Cameroon	mf	60	M	40	2	+	FR3
MC 326	Cameroon	mf	46	M	45	2	+	FR3
MC 328	Cameroon	mf	37	M	74	6	+	FR3
MC 331	Cameroon	mf	54	M	78	5	+	FR3
MC 333	Cameroon	mf	43	M	300	6	+	FR3
MC 335	Cameroon	mf	23	M	30	4	+	FR3
MC 341	Cameroon	mf	22	M	52	5	+	FR3
MC 343	Cameroon	mf	35	M	200	2	−	FR3
MC 352	Cameroon	mf	60	M	200	14	+	FR3
MC 360	Cameroon	mf	66	F	98	6	+	FR3
MC 362	Cameroon	mf	70	F	26	6	+	FR3
Ab-E11955	Southern Africa	HC	17	F	0	0	−	TS
Ab-E11962	Southern Africa	HC	17	M	0	0	−	TS
Ab-E11968	Southern Africa	HC	17	F	0	0	−	TS
Ab-E11969	Southern Africa	HC	17	F	0	0	−	TS
Ab-E11970	Southern Africa	HC	17	F	0	0	−	TS
Ab-E12152	Southern Africa	HC	25	M	0	0	−	TS
Ab-E12153	Southern Africa	HC	33	M	0	0	−	TS
Ab-E12160	Southern Africa	HC	47	M	0	0	−	TS

*Nod.*, nodule positive, *NEC* non-endemic control, *mf* microfilaridermic, *HC* healthy control, *KCCR* Kumasi Centre for Collaborative Research, *FR3* Filariasis Research Reagent Resource Center, *TS* Tissue Solutions, Ltd.

A second set of EDTA plasma samples was collected as part of a field study in Ghana. This study was undertaken in an onchocerciasis-endemic community located in Adansi South District along the Pra River basins in the Ashanti Region of Ghana. This study was approved by the Committee on Human Research Publications and Ethics of the School of Medical Sciences of the Kwame Nkrumah University of Science and Technology, Kumasi, Ghana, and study subjects signed an informed consent form. Physical examinations were performed to identify those subjects having palpable nodules. Skin snips (biopsies) were then taken from those with nodules in order to determine the microfilarial (mf) load in the skin (Debrah et al. 2015). Most subjects were participating in MDA programs. A total of 23 nodule-positive subjects that donated plasma samples were included. Additionally, EDTA plasma samples from six non-endemic controls (from Kumasi, Ashanti Region) were included. An overview of the patient demographics is also provided in Table 1. All samples were stored at −80 °C until analysis.

### Synthetic microRNA molecules and generation of miRNA standard curves

Six RNase-free 5′-phosphorylated miRNA oligoribonucleotides were synthesized (Integrated DNA Technologies) for the validation of the miRNA assays, corresponding to bma-miR-71, ov-miR-71 22nt, ov-miR-71 23nt, ov-miR-87-3p, hsa-miR-425-5p, and hsa-miR-93-5p. Stock solutions of 100 μM synthetic oligonucleotide in RNase-free and DNase-free water were prepared according to the quantities quoted by the manufacturer (based on spectrophotometric analysis). Tenfold dilution series were prepared in RNase-free and DNase-free water, starting at 10^8^ copies/μL down to 10^2^ copies/μL. These samples and no template control (NTC; zero copies) were further processed and analyzed similar to the study samples.

### miRNA analysis by miRCURY LNA Universal RT miRNA PCR

RNA was purified from 25 or 200 μL serum/plasma, as indicated, with the miRNeasy kit from Qiagen (Venlo, Holland) according to the manufacturers’ protocol. Reverse transcription reactions were performed using the miRCURY LNA Universal RT MicroRNA PCR Universal complementary DNA (cDNA) kit II (Exiqon, Vedbaek, Denmark). Reverse transcription thermocycling parameters were as follows: 42 °C for 60 min and 95 °C for 5 min. qPCR was performed using the miRCURY LNA Universal RT microRNA PCR system (Exiqon, Vedbaek, Denmark) with 17 parasitic miRNAs and 2 human miRNAs as internal control. All primer/probe sets for parasitic miRNAs were custom designed by the supplier. Also, three extraction controls and two cDNA synthesis controls were used as indicated by the provider. All miRNAs analyzed are listed in Table 2. Two real-time qPCR amplifications were performed for each RT reaction. Reactions were performed according to the manufacturers’ instructions using a LightCycler 480 system (Roche). qPCR thermocycling conditions were as follows: 95 °C for 10 min, followed by 45 cycles of 95 °C for 10 s and 60 °C for 1 min. Melt curve analysis was performed between 60 and 95 °C at a ramp rate of 0.11 °C/s. Detectable miRNAs were those with a Cq (quantification cycle) <40.0 in one of both duplicates. An overview of the different sample volumes used for extraction, RNA volumes used for cDNA synthesis, and the different cDNA dilutions used for qPCR is presented in Table 3.

**Table 2:** miRNAs analyzed in this study

miRNA	Sequence	Remark	Ref.
bma-miR-36a	TCACCGGGTGCACATTCGGTC	Female specific	Poole et al. (2014)
bma-miR-84	TGAGGTAGTTTATAAAGCTGCGA	Adult specific	Poole et al. (2014)
bma-miR-5364	CGAGGTATTGTTTATTGGCTGA	Adult specific	Poole et al. (2014)
bma-miR-236-1	TAATACTGTCAGGTAATGACGAT	Adult specific	Poole et al. (2014)
bma-miR-71 21nt	TGAAAGACATGGGTAGTGAGA	mf specific	Poole et al. (2014)
ov-miR-71 23nt	TGAAAGACATGGGTAGTGAGACG	Found in human serum	Quintana et al. (2015)
ov-miR-71 22nt	TGAAAGACATGGGTAGTGAGAC	Found in human serum	Quintana et al. (2015)
ls-miR-86	TAAGTGAATGCTTTGCCACAGTCT	Found in mouse serum	Buck et al. (2014)
ls-miR-263	AATGGCACTAGATGAATTCACGG	Found in mouse serum	Buck et al. (2014)
ls-miR-100a/ov-miR-100a	TACCCGTAGCTCCGAATATGTGT	Found in mouse and human serum	Buck et al. (2014); Quintana et al. (2015)
ls-miR-100b/ov-miR-100d	AACCCGTAGTTTCGAACATGTGT	Found in mouse and human serum	Buck et al. (2014); Quintana et al. (2015)
ls-miR-100c	AACCCGTAGAATTGAAATCGTGT	Found in mouse serum	Buck et al. (2014)
ov-miR-87-3p	GTGAGCAAAGTTTCAGGTGTTC	Found in human serum	Quintana et al. (2015)
ls-bantam-a/ov-bantam-a	TGAGATCATTGTGAAAGCTATT	Found in mouse and human serum	Buck et al. (2014; Quintana et al. (2015)
ls-bantam-b	TGAGATCACGTTACATCCGCCT	Found in mouse serum	Buck et al. (2014)
ls-bantam-c	TGAGATCATGCCACATCCGTCT	Found in mouse serum	Buck et al. (2014)
ov-lin-4	TCCCTGAGACCTCTGCTGCGA	Found in human serum	Quintana et al. (2015)
hsa-miR-425-5p	AATGACACGATCACTCCCGTTGA		
hsa-miR-93-5p	CAAAGTGCTGTTCGTGCAGGTAG		
UnsiSp2	Extraction spike-in		
UnsiSp4	Extraction spike-in		
UnsiSp5	Extraction spike-in		
UnsiSp6	cDNA synthesis spike-in		
cel-miR-39-3p	cDNA synthesis spike-in

**Table 3:** Variable RNA input and cDNA dilutions used for different sample sets

Sample set	Sample input (μL)	RNA input (μL)	cDNA dilution	LOD^a^ (log copies/μL plasma)
KCCR	200	5	1/50	1.64
KCCR	200	5	1/8	0.85
FR3 + TS	25	6	1/50	2.46

*KCCR* Kumasi Centre for Collaborative Research, *FR3* Filariasis Research Reagent Resource Center, *TS* Tissue Solutions, Ltd.

^a^Based on 28 copies/qPCR reaction and 100% extraction and cDNA synthesis efficiency

### Statistical analysis

Results of internal and external controls (extraction controls, cDNA synthesis controls, and human reference miRNAs) were used as quality control of the samples evaluated. Results of these controls were subjected to outlier testing in order to identify samples with poor extraction and/or cDNA synthesis efficiency. For each control assay, all samples analyzed under the same conditions (i.e., same input volume and cDNA dilution) were subjected to outlier detection with a Grubbs’ test (using a significance level of 0.05) using the GraphPad online outlier calculator (http://graphpad.com/quickcalcs/Grubbs1.cfm) (Grubbs 1969). In case an outlier was detected, sample was not included for further analysis. This process of outlier identification was iterated until no outliers were detected. For comparison of Tm values in different groups, two-tailed unpaired *t* test was performed using the GraphPad Prism version 6.02. *P* value <0.05 was considered to be significantly different.

## Results

### Assay validation: standard curves and cutoff determination

**Fig. 1:**
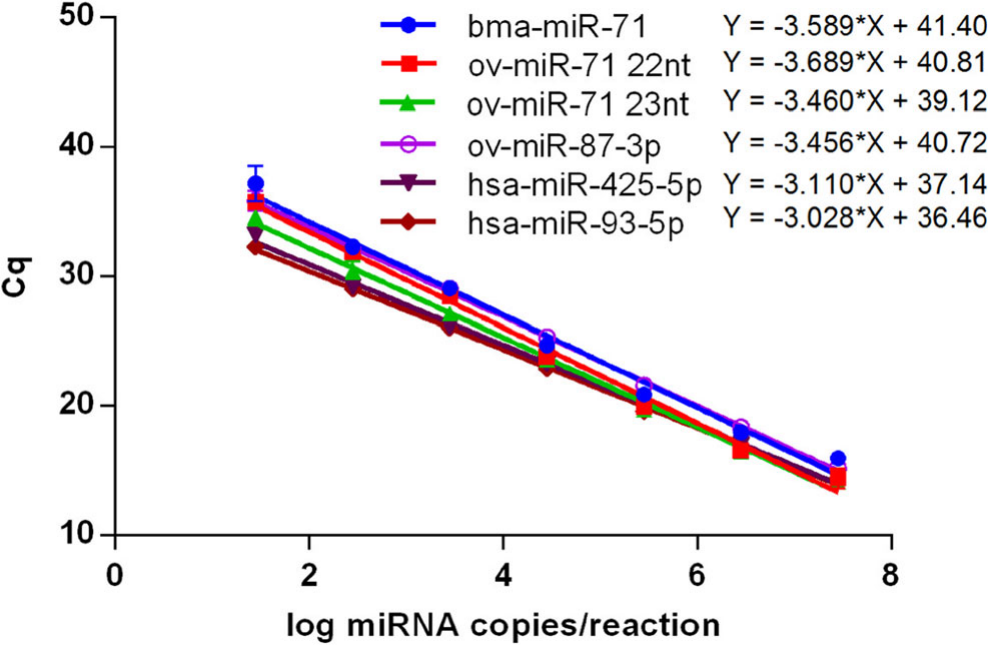
Standard curves of six synthetic miRNAs

In order to confirm the performance of the miRCURY LNA Universal RT microRNA PCR system, dilution curves were prepared for four parasitic miRNAs (bma-miR-71, ov-miR-71 22nt, ov-miR-71 23nt, and ov-miR-87-3p) and two human reference miRNAs (hsa-miR-425-5p and hsa-miR-93-5p). Each dilution curve was analyzed in duplicate using its respective assay, and resulting Cq values were used to generate standard curves (Fig. 1). All tested assays had good linearity (*r* ^2^ > 0.98) and acceptable PCR efficiency (slope was between −3.12 and −3.89, corresponding to PCR efficiencies between 80.8 and 109%). The lowest concentration used for each miRNA, 28 copies/reaction (1.45 log copies/reaction), was well detectable in all assays. The average Cq value for the four *O. volvulus* miRNAs at this concentration, being 35.9, was therefore chosen as cutoff for positivity in all miRNA assays used in this work. This cutoff approximates the cutoff of 37, recommended by the supplier. Based on this cutoff, for each experimental setup, a limit of detection (number of copies/μL plasma) was calculated and ranged between 0.85 and 2.46 log copies/μL plasma (Table 3).

### Determination of plasma levels of parasitic miRNAs in samples from nodule-positive subjects

Based on the information that has been published before, a list of 17 parasitic miRNAs was compiled (Buck et al. 2014; Poole et al. 2014; Quintana et al. 2015). A number of these miRNAs were found upon small RNA sequencing of serum samples from *O. volvulus*-infected individuals, while others were detected for other filarial species and have homologous sequence in *O. volvulus* (Table 2). For each of these miRNAs, a PCR primer set was designed (Exiqon, Vedbaek, Denmark) and used to analyze a set of plasma samples collected from 23 nodule-positive individuals and 6 non-endemic controls living in Ghana.

**Fig. 2:**
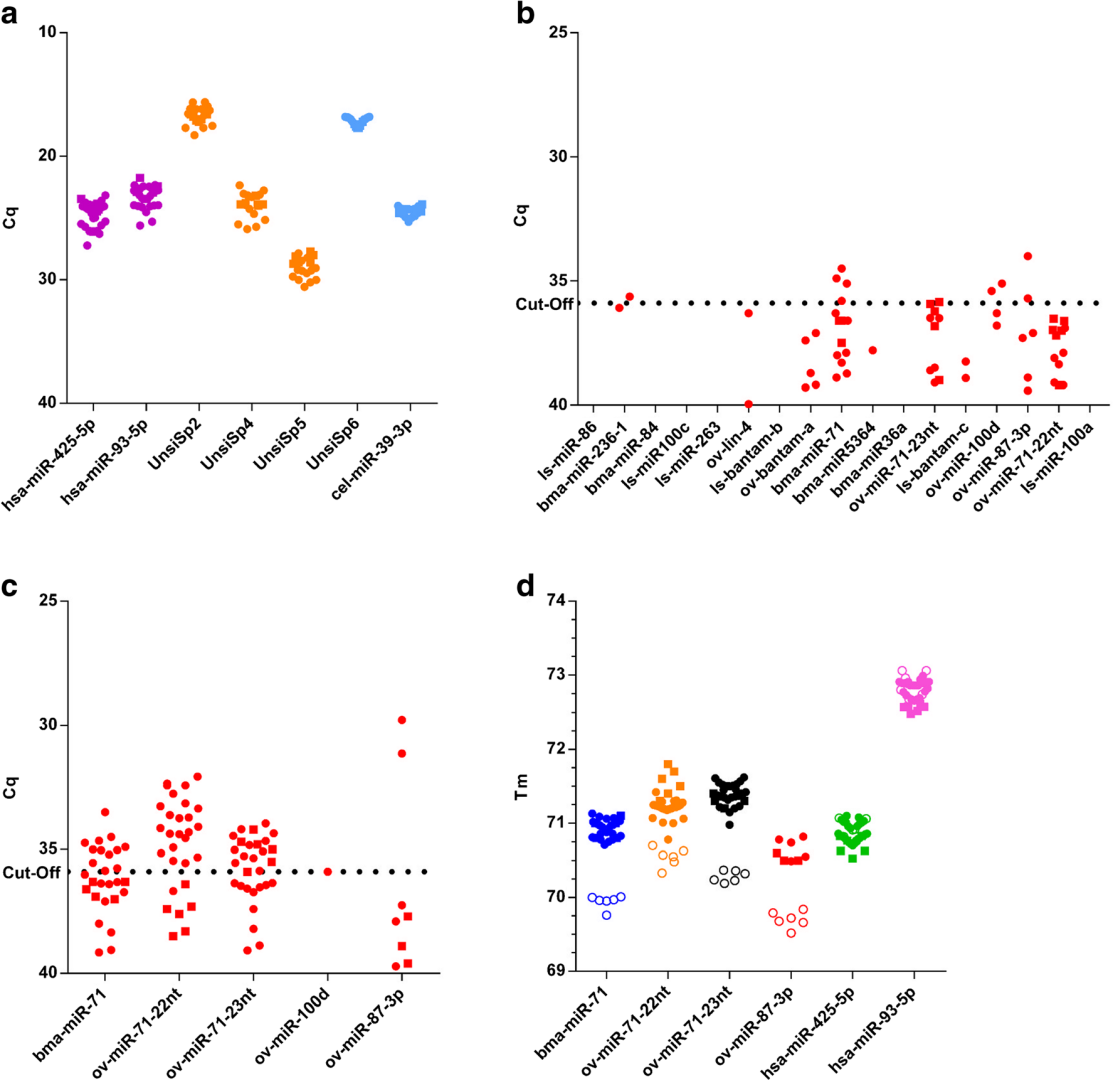
miRNA analysis in plasma from nodule-positive individuals (*closed circles*) and non-endemic controls (*closed squares*) from Ghana. **a** Internal and external control results. **b** Results of 17 parasitic miRNAs. **c** Result of five selected *O. volvulus* miRNAs, using less diluted cDNA. **d** Tm determination of miRNA amplicons obtained with clinical samples (*closed circles*) and synthetic miRNAs (*open circles*)

Results of internal and external controls were similar for all samples (Fig. 2a). Of the 17 parasitic miRNAs analyzed, only 5 appeared to be found positive in a number of samples: bma-miR-236-1, bma-miR-71, ov-miR-71-23nt, ov-miR-100d, and ov-miR-87-3p (Fig. 2b). Since it is known that less diluted cDNA is well tolerated in the miRCURY LNA Universal RT microRNA PCR system, we investigated whether sensitivity of the qRT-PCR assays could be increased by using less diluted cDNA (Egidi et al. 2015; Li et al. 2014). Therefore, samples were reanalyzed for the three miR-71 variants, ov-miR-100d, and ov-miR-87-3p, using 8-fold diluted cDNA (Fig. 2c). Most samples now appeared to show positive signals for bma-miR-71, ov-miR-71-22nt, and ov-miR-71-23nt. For ov-miR-87-3p, 2 samples turned out to be positive, one more than using more diluted cDNA. Of the two positive results found for ov-miR-100d, only one could be confirmed. In order to confirm the specificity of the signals observed for the miR-71 variants and ov-miR-87-3p, melting curves obtained with the synthetic miRNAs and all clinical samples that had signal in qRT-PCR (also those with Cq above the cutoff) were compared (Fig. 2d). Even though both sample sets were analyzed using the same qPCR conditions, a highly significant difference in Tm values was appreciated (*P* < 0.001), indicating that the signals obtained for these miRNAs in the clinical samples are not specific and are presumably caused by non-specific amplification of one or more other miRNAs present in the samples. Results are shown for cDNA that was diluted 8-fold, but melting temperatures obtained using 50-fold diluted cDNA showed similar differences in Tm values (data not shown). In contrast, Tm values obtained for the human reference miRNAs did perfectly match between the synthetic miRNAs and clinical samples (*P* > 0.05).

### Determination of serum levels of parasitic miRNAs in samples from microfilaridermic subjects

**Fig. 3:**
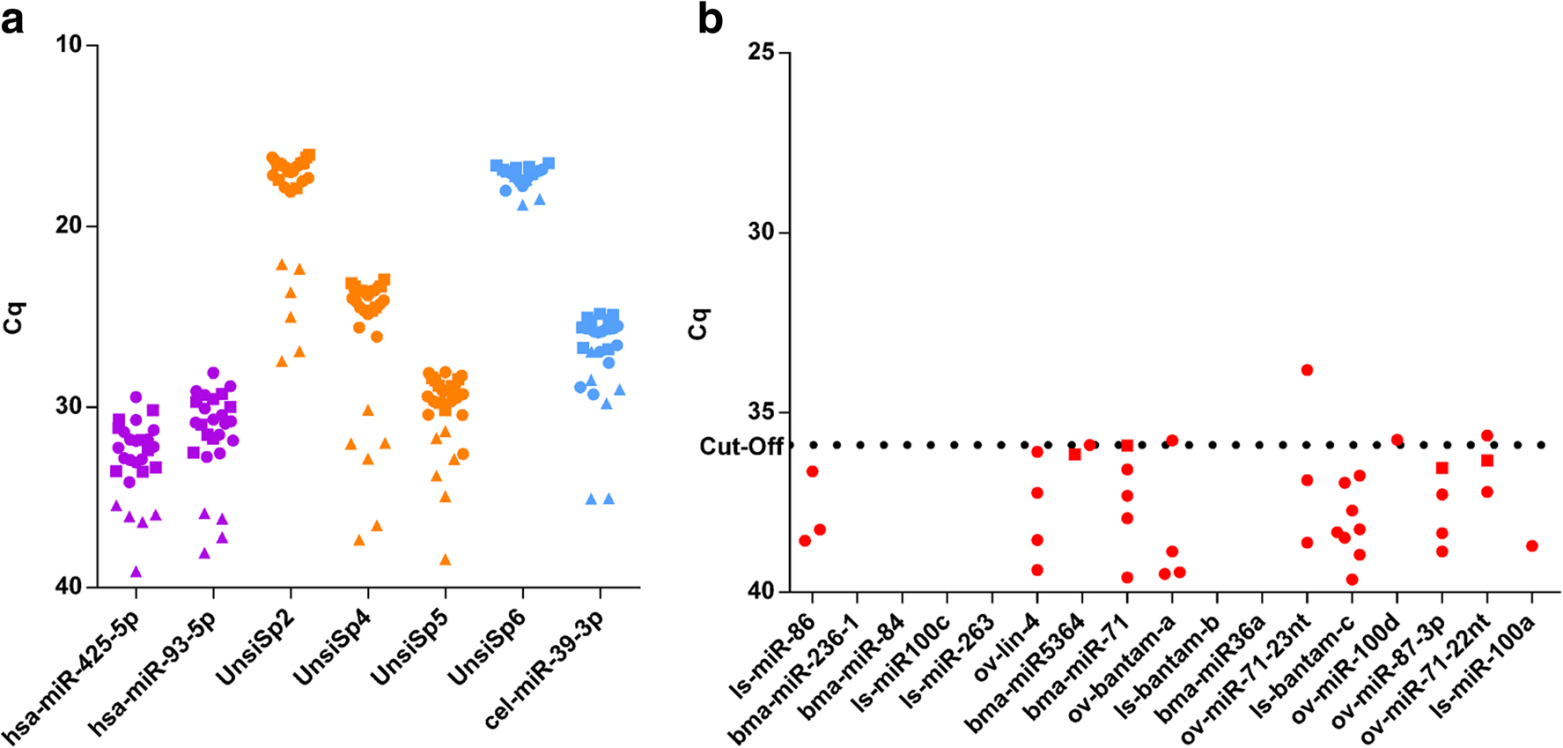
miRNA analysis in serum from individuals with high microfilarial load from Cameroon (*closed circles*) and healthy controls from Southern Africa (*closed squares*). **a** Internal and external control results. Samples that showed reduced extraction and/or cDNA synthesis are indicated by *triangles*. **b** Results of 17 parasitic miRNAs

We also determined the levels of these miRNAs in serum from 20 individuals with high microfilarial load (>25 mf/mg) and 6 healthy controls. All individual results of internal and external controls are graphically summarized in Fig. 3a. Based on the different spike-ins, six samples from infected individuals out of 20 were excluded from further analysis as they showed a markedly poorer extraction efficiency and/or cDNA synthesis efficiency and were identified as outliers (samples MC260, MC211, MC224, MC234, MC253, and MC215). These samples also had lower levels of human miRNAs, confirming their poor extraction and/or cDNA synthesis efficiency. All samples from healthy volunteers had good extraction and cDNA synthesis efficiency, as well as relatively high expression of human miRNAs. When comparing the results of the human miRNAs in these serum samples with the plasma samples described previously, a substantial difference is observed. This is first of all attributed to the fact that lower input volumes have been used during extraction of these serum samples (Table 3). Secondly, it is known that plasma and serum miRNA levels do not fully correlate and plasma levels tend to be slightly higher (according to the guidelines for the miRCURY LNA Universal RT microRNA PCR system).

Results of the parasitic miRNAs of all samples that passed previous selection are presented in Fig. 3b. Only 4 of the 17 parasitic miRNAs investigated showed a positive signal in one of the samples analyzed (ov-bantam-a, ov-miR-71-23nt, ov-miR-100d, and ov-miR71-22nt). For the other parasitic miRNAs, signals were detected, but signals observed were very low and, in many cases, were present both in samples from infected individuals and healthy volunteers.

## Discussion

The discovery of miRNAs being secreted by parasites into the circulation of the host has resulted in several speculations about their diagnostic utility. Whereas for blood-borne parasites, such as *Lo. loa*, *D. immitis*, and *Li. sigmodontis*, large number of different miRNAs was detected in the host circulation, initial studies on plasma or serum from *O. volvulus*-infected individuals only revealed a very small number of parasite-derived miRNAs (Buck et al. 2014; Quintana et al. 2015; Tritten et al. 2014a; Tritten et al. 2014b). Also, the amount of these miRNAs was very low, already indicating a sensitivity challenge for its diagnostic utility. This sensitivity challenge becomes even more important when looking to the possibility of using parasite-derived miRNAs for treatment monitoring, as a sufficiently broad dynamic range is required for this application.

We have used standard miRNA extraction and RT-qPCR technology to detect 17 of these previously identified miRNAs in plasma or serum from *O. volvulus*-infected individuals. A first sample set was collected from nodule-positive individuals in Ghana with very low or no microfilaria detected in the skin snip, while a second sample set was obtained from nodule-positive individuals in Cameroon with high microfilarial load. When both datasets are combined, only seven miRNAs appeared to result in a detectable signal in qPCR: bma-miR-236-1, bma-miR-71, ov-miR71-22nt, ov-miR-71-23nt, ov-miR-100d, ov-bantam-a, and ov-miR-87-3p. Interestingly, all except bma-miR-236-1 were previously also detected in serum from infected individuals (Quintana et al. 2015). For four of these parasitic miRNAs (three miR-71 variants and ov-miR-87-3p), calibration curves were prepared using synthetic miRNAs and Tm was determined both for these synthetic miRNAs and the clinical samples. For all four parasitic miRNAs, Tm differed significantly between the clinical samples and the synthetic miRNAs, while a similar exercise for the human reference miRNAs showed no difference. This observation strongly suggests that the signals observed for these parasitic miRNAs are caused by non-specific amplification and are not reflecting true positive signals. Furthermore, as was also observed previously, plasma levels of the other miRNAs are extremely low with Cq values just below the cutoff. Also, these miRNAs were detected in only very few samples and are not universally present in all or most infected individuals, which is an essential property for use as a diagnostic marker.

The discrepancy in Tm between synthetic miRNAs and clinical samples highlights the importance of using synthetic miRNAs to confirm the specificity of the RT-qPCR signals obtained. Whereas in small RNA sequencing technologies, the sequence of every single miRNA molecule is determined, qPCR-based detection methodologies are sensitive to non-specific detection (Dellett and Simpson 2016). Since miRNAs are usually only 18–22 nucleotides in length, specificity remains one of the key issues when developing RT-qPCR assays to measure microRNAs for molecular diagnosis (Bartel 2004; Dellett and Simpson 2016). Especially when analyzing low abundant miRNAs in a background of millions of copies of other miRNAs (and other small RNAs), one needs to confirm the specificity of the assays before drawing any conclusion.

Importantly, our study does not completely exclude the applicability of plasma-derived parasitic miRNAs as a tool to detect the presence of parasite using minimally invasive sampling procedures. Our work indicates that sensitive methodologies will be required that also demonstrate high specificity. As this study was limited to only one qPCR-based technology using commercially available reagents, further optimization of the qPCR assays or evaluation of other detection platforms, such as stem-loop RT-PCR, might offer some improvements (Chen et al. 2005; Mestdagh et al. 2014). It remains, however, questionable whether detection of parasitic miRNAs with sufficient diagnostic power will be feasible, even after elaborate assay optimization. The most obvious path forward to increase sensitivity might be to use higher volumes of plasma for miRNA extraction. Standard miRNA extraction kits, however, are not designed to use higher volumes, and this approach would therefore have limited feasibility in practice. Alternatively, a preamplification step might be introduced as this has been shown before to boost sensitivity (Tan et al. 2015). One other interesting approach might be the use of a precursory isolation of parasite-specific exosomes. It was shown for the helminth parasites *Heligmosomoides polygyrus* and *B. malayi* that these parasites secrete miRNA-containing vesicles, possibly involved in modulation of the host’s immune response (Buck et al. 2014; Zamanian et al. 2015; Zheng et al. 2016). Isolation of total exosomes or even of parasite-specific exosomes could result in an enrichment of parasitic miRNAs, thereby reducing the background of non-parasite-derived miRNAs during analysis. The same work, however, also indicated that exosomes and associated RNAs operate locally in the host’s body and that their detection will depend on the stage and localization of the parasite in the host (Buck et al. 2014). It is therefore not surprising to observe the low amounts of parasitic miRNAs in the circulation upon infection with the skin localized *O. volvulus* as compared to, e.g., infection with *D. immitis* or *B. malayi*, two parasites with a blood-borne microfilarial stage (Tritten et al. 2014a).

In conclusion, our data indicate that *O. volvulus* miRNAs are not present in circulation in sufficiently high amounts to be used as a diagnostic marker for infection or treatment monitoring using LNA-based RT-qPCR analysis.

Cq, Quantification cycle; mf, microfilaria; miRNA, microRNA; *O. volvulus*, *Onchocerca volvulus*; qPCR, quantitative PCR; RT, reverse transcriptase
